# Addressing the disposal of unused and expired medications in Saudi households: Time to bridge the regulatory gaps

**DOI:** 10.1371/journal.pone.0350117

**Published:** 2026-06-16

**Authors:** Maryam Farooqui, Aseel Alrashed, Duaa Alfunayyikh, Raffal Alassaf, Reem Aldubayan, Saud Alsahali, Wan Ismahanisa Ismail, Suhaj Abdulsalim

**Affiliations:** 1 Department of Pharmacy Practice, College of Pharmacy, Qassim University, Buraydah, Saudi Arabia; 2 Discipline of Social and Administrative Pharmacy (DSAP), School of Pharmaceutical Sciences, Universiti Sains Malaysia (USM), Gelugor, Pulau Pinang, Malaysia; 3 Faculty of Health Sciences, Universiti Teknologi MARA (UiTM), Cawangan Pulau Pinang, Kampus Bertam, Pulau Pinang, Malaysia; Mizan-Tepi University, ETHIOPIA

## Abstract

**Introduction:**

This study aimed to investigate the awareness, and practice towards disposal of unused, leftover, and expired medications among the residents of Qassim, Saudi Arabia.

**Materials and methods:**

A cross-sectional survey was conducted among 877 residents through an online-based questionnaire Participants have to be adults aged 18 years or older of either gender, psychologically stable, able to provide informed consent and understand the Arabic language otherwise, were excluded. A pre-validated, structured questionnaire was used for data collection. Data was analyzed using R software. For all analyses, p < 0.05 was considered significant.

**Results:**

Most of the [70%] respondents had unused medications, [62%] reported to store their unused medications in the refrigerator followed by bedroom cabinet [36%]. The majority [88%] dispose of unused medicines by throwing away in the household dustbins. The vast majority throw away near expiry [73%], and expired medicines [92%], in the household dustbins. Majority [86%] of the participants denied receiving formal education/awareness regarding safe medications disposal where [60%] agrees that the responsible sector for educating the public about safe medications disposal should be pharmacies. A significant but weak association [p < 0.05; φc = 0.1], between the storage of unused medicines at home and demographic factors such as age, marital status, education, and income was found.

**Conclusions:**

In conclusion our study highlighted the concern that study respondents frequently store medications in refrigerator [62%] and discard unused and expired medications improperly in the dustbin [88%]. Study results call upon concerned authorities to raise awareness and provide regulatory guidelines for the safe disposal of medications by the residents.

## Introduction

Medications are the main component of medical waste, which increases with higher consumption [1]. Patients often cannot use all dispensed medications due to non-compliance, therapy changes, adverse effects, promotional practices, and prescribing habits such as polypharmacy. Other factors include easy access to OTC medications, improved conditions leading to treatment discontinuation, or death [2,3]. Medication wastage is a concern due to its financial burden and negative impacts on health and the environment. Unused medications pose risks of diversion, abuse, and accidental overdose, arising from households or healthcare settings [2]. Emphasis on rational medicine use ensures patients receive and use appropriate medications [4]. Waste medications are defined as any substance or object discarded or required to be discarded. The WHO characterizes medical waste as waste generated in diagnosing, treating, or immunizing humans or animals [4].

Medicines are crucial for treating many diseases, but proper disposal at the end of treatment is critical. Knowledge and awareness of drug disposal are essential for a safe environment. Lack of knowledge can lead to environmental pollution and health hazards [5]. Urgent action is needed to address improper medicine disposal [6]. Medications can enter the environment through inadequate disposal in sinks, toilets, or household waste, as well as human excretion [1]. Inappropriate disposal can contaminate surface and drinking water, as sewer systems are not equipped to eliminate medicines efficiently, potentially increasing medication resistance and causing long-term genetic effects in humans and marine life [1,7].

The accumulation of unused medications leads to medication waste and economic resource losses. Many economic losses could have been avoided with appropriate measures, benefiting other healthcare schemes. Medication wastage is a universal issue in developed countries. For instance, annual drug wastage is about £300 million in England, while consumers in the US wasted over $418 billion in 2012 due to suboptimal medicine use [8]. A study found an average of 2.2 [SD 2.7] expired, unused, or deteriorated home medications per household in Saudi Arabia and 2.7 [SD 1.9] in other Gulf countries [9]. Estimated medication waste was 25.8% in KSA and 41.3% in other Gulf countries, with ~$150 million spent on unused medications [9]. Unfortunately, no recent publications were found in KSA [10].

Many studies have investigated the knowledge about medication waste in general population in various countries. In the USA, about 59% of respondents disposed of medications in household garbage, and 31% flushed them down in the toilet or sink, with over 80% stating they had never received information about proper disposal [11]. In India, 85.4% kept unused medicines at home, and 84% discarded expired medicines into the dustbins, while only 6% returned them to pharmacies, and 16% kept expired medicines [12]. In Kuwait, the most common disposal methods were throwing medicines in the trash [76.5%] or flushing them [11.2%], with 8.5% giving them to friends and 11.9% returning them to pharmacies [7]. In Saudi Arabia, a study in Jeddah found that 91.57% discarded expired medications in household garbage, and 2.98% returned them to hospitals or pharmacies [13]. Another study in Riyadh showed that 65% kept medicines until they expired, 48.1% threw them away, and 13.7% gave them to friends or relatives; only 5.4% returned them to medical stores [14]. In Saudi Arabia, lack of standardized disposal rules or collecting mechanisms, as well as a lack of knowledge about appropriate pharmaceutical disposal techniques exacerbate these issues. Despite the growing concern over the safe handling of unwanted and expired medications, research on the disposal habits and attitudes of the Saudi populations is lacking. This study aims to investigate the disposal behaviors, awareness levels, and influencing factors among the Qassim region of Saudi Arabia.

## Materials and methods

### Study design and population‌‌

A questionnaire-based, cross-sectional study was conducted at Al-Qassim region, starting from 20 December 2022 to 28 February 2023. At the time of data collection, Al-Qassim region has a population of 1215660 [15]. Because of its agricultural assets, it is known as Saudi Arabia’s “alimental basket.” It has over 400 cities, towns, villages, and Bedouin settlements, with ten of them represented as governorates. Convenience sampling was adopted, where participants were recruited through online dissemination of the questionnaire via personal and institutional networks. The participants have to be adults aged 18 years or older of either gender, able to provide informed consent and understand the Arabic language. Individuals who don’t meet the inclusion criteria were excluded.

### Sample size

By using Raosoft software [16], the sample size was calculated. Using a confidence interval of 95%, a margin of error of 5%, and a target population of 1215660 residents of Al-Qassim region, the minimum required sample size was 377 participants.

### Study tool

A 64-item questionnaire consists of four sections was used, i] demographic information, ii] assessment of practice toward storage and disposal practices of unused medicines, iii] assessment of the practices and disposal of expired medicines and the fourth section, iv] assessment of awareness of medication disposal. We conducted a thorough review of the literature [17–19] and created the first version of the questionnaire in English. Besides the demographic information, the questionnaire has assessed awareness and practices regarding unused and expired medicines. The forward-backward method was used to translate the questionnaire into Arabic by bilingual experts. The internal consistency was established using Cronbach’s alpha which was 0.8 and was acceptable as per the literature. For face and content validity, the questionnaire was reviewed by experts and was pre-tested on 30 respondents. Minor changes have been highlighted during the pilot study. Data from the pilot phase was not utilized for final analysis [20].

### Data collection technique

Data was collected through an online-based questionnaire. Before commencing the survey, each participant was thoroughly informed about the aims and objectives of the study. Participants were explicitly informed that their involvement in the study was entirely voluntary. The survey was estimated to take approximately 5–6 minutes to be completed.

### Data analysis

Completed questionnaires were reviewed for completeness and consistency prior to analysis. Questionnaires with missing responses were excluded from the analysis. “Not sure” responses were treated as a separate category and were excluded from the denominator when calculating percentages for knowledge-based questions. Logical checks were applied to identify inconsistent responses, and any identified discrepancies were reviewed and corrected where possible. Data was coded and added to R software for analysis. Subsequently, descriptive analysis was carried out based on the study’s objectives. The categorical variables were presented by frequency and percentage, to analyze associations, the Chi-Square test was used, and Phi/Cremer was used to measure association between two nominal variables that were applicable. For all analyses, p < 0.05 is considered as significant.

### Ethical consideration

This research was conducted on humans according to the guidelines of the Declaration of Helsinki. The study received approval from the Qassim Ethics Committee [Approval no: 607\44\7974]. Informed consent was obtained electronically via a consent checkbox after participants reviewed the online information sheet; participation was voluntary and no personal identifiers were collected.

## Results

### Demographic characteristics of the participants

Table 1 summarizes the demographic features of survey participants. The survey received 877 responses, most of them were female [81%], with 34% of the respondents ranging in age from 40 to 54 years, most respondents [77%] hold a graduate degree, residents of urban areas make up nearly all survey participants [98%]. More than half of the participants [65%] had incomes between 5000 SR and 20000 SR, while only 8.9% earned more than 20000 SR.

**Table 1:** Demographic characteristics of the participants.

Characteristic	n=877 [%]
**Gender**	
Male	166 [19%]
Female	711 [81%]
**Age [years]**	
18-24	191 [22%]
25-39	237 [27%]
40-54	301 [34%]
55-69	140[16%]
70-85	8 [1%]
**Marital status**	
Single	294 [34%]
Married	583 [66%]
**Family size**	
≤ 5	358 [41%]
> 5	519 [59%]
**Residency**	
Urban	860 [98%]
Rural	17 [1.9%]
**Education**	
Primary	10 [1.1%]
Secondary	21 [2.4%]
Higher secondary	171 [19%]
Graduate	675 [77%]
**Monthly income in riyal**	
No income to 5000SR	231 [26%]
50001 SR-1000SR	230 [26%]
100001 SR-20000 SR	338 [39%]
More than 20000 SR	78 [8.9%]

Data are presented as number [%].

### Storage, and disposal practices of unused medicines

Table 2 shows that 70% of the people who filled out the survey had unused medications. 62% of those people store their unused medications in the refrigerator, followed by the bedroom cabinet [36%]. In addition, 77% said they keep medicines because they could be used again, and 55% said they keep them because they might be needed in an emergency, the majority [88%] dispose of unused medicines stored at your home by throw away in dustbins [household trash], 68% and 62% answered that they know how to store medicines at home and read storage instructions on the labels/leaflets respectively.

**Table 2:** Storage, and disposal practices of unused medicines.

Characteristic	n=877 [%]
**Do you currently have any unused medicines?**	
No	177 [20%]
Yes	612 [70%]
Not sure	88 [10%]
Where do you store your unused medicine? *(n = 612)	
Refrigerator	379 [62%]
Kitchen cabinet	88 [14%]
Bedroom cabinet	222 [36%]
Medicine cabinet	179 [29%]
Use more than one place for storage	204[33%]
Why do you keep unused medicines at home? *(n = 612)	
Can reuse the medicine	472 [77%]
Medicine is needed in an emergency	334 [55%]
Can be used by friends or family members	80 [13%]
**Do you dispose unused medicines stored at your home? (n = 612)**	
No	77 [13%]
Yes	162 [26%]
Sometimes	373 [61%]
How do you dispose of unused medicines stored at your home? *(n = 612)	
The exchange at the pharmacies	55 [9.0%]
Throw away in dustbins [household trash]	537 [88%]
Give to friends or relatives	94 [15%]
**Do you know how to store medicines at home?**	
No	279 [32%]
Yes	598 [68%]
**Do you read storage instructions on the labels/leaflets?**	
No	336 [38%]
Yes	541 [62%]
**Do you check the expiry date of the medicines before purchasing?**	
No	288 [33%]
Yes	589 [67%]
**Do you check the expiry date before using the medicines?**	
No	105 [12%]
Yes	772 [88%]
**Do you know the procedure to disposal of nearly expired medicines? [Nearly expired means a month to expire]**	
No	541 [62%]
Yes	336 [38%]
How do you dispose of nearly expired medicines? *	
Donate to hospital/ clinic	72 [8.2%]
Return it to pharmacies	80 [9.1%]
Throw away in dustbins [household trash]	644 [73%]
Flush in toilet or sink	89 [10%]
Give them to friends/family members/ others	93 [11%]
Keep at home until expired	324 [37%]
**Do you know the procedure to dispose of expired medicines?**	
No	483 [55%]
yes	394 [45%]
How do you dispose expired medicines? *	
Throw away in dustbins [household trash]	809 [92%]
Flush in toilet or sink	95 [11%]
Return them to pharmacies or hospitals for disposal	39 [4.4%]
No action [keep at home]	74 [8.4%]

* denoted that the question may had more than one answer and the percentage could be more than 100%.

Regarding checking the expiration date, 67% do so before purchasing, and 88% do that before using the medication**,** only 45% know the procedure to dispose of expired medicines, many people throw away near expiry and expired medicines [73 percent and 92%, respectively] in dustbins [household trash].

### Practices and disposal of expired medicines

Table 3 shows that 86% of the survey participants did not receive education regarding safe disposal of medications, of those 60% denied that the responsible sector for educating the public about safe disposal of medications are the pharmacies followed by social media [46%], 33% and 31% are strongly agree and agreed with unsafe disposal of medications associated with harmful consequences for public health and the global environment.

**Table 3:** Practices and disposal of expired medicines.

Characteristic	n=877 [%]
**Have you received education regarding safe disposal of medications?**	
No	757 [86%]
yes	120 [14%]
**Do you seek information from official resources regarding safe disposal of medications?**	
Always	368 [42%]
Often	162 [18%]
Sometimes	196 [22%]
Rare	66 [7.5%]
Never	85 [9.7%]
What are responsible sectors to educate the public about safe disposal of medications?*	
Hospitals	390 [44%]
Pharmacies	527 [60%]
Social media	407 [46%]
Medical waste companies	390 [44%]
**In your opinion, are unsafe disposal of medications associated with harmful consequences on public health and global environment?**	
Strongly disagree	6 [0.7%]
Disagree	19 [2.2%]
Don’t know	291 [33%]
Agree	270 [31%]
Strongly agree	291 [33%]
**Do you agree that the presence of designated medication disposal locations or medications disposal process will improve safe medication waste management practices?**	
Strongly disagree	2 [0.2%]
Disagree	7 [0.8%]
Don’t know	83 [9.5%]
Agree	273 [31%]
Strongly agree	512 [58%]
**Do you agree that locating medications disposal facilities via smart phone application would assist in safe drug disposal practice?**	
Strongly disagree	4 [0.5%]
Disagree	14 [1.6%]
Don’t know	93 [11%]
Agree	326 [37%]
Strongly agree	440 [50%]
What are the effective methods to raise the community awareness regarding medications disposal?*	
Deliver clear instructions while patients receive medications raise community awareness	658 [75%]
Patient education by health care professionals	458 [52%]
Public awareness via social media	553 [63%]
Other methods [e.g., Write on medication package, giving educational curriculum]	17[1.9%]
What is the best method to dispose medication from participants’ point of view?*	
Return to pharmacy	431 [49%]
Take it to the hospital	258 [29%]
Through household trash	418 [48%]
Through toilet flush	95 [11%]
Other	32[3.4%]
Your opinion about disposing unused/expired medications?*	
All medications must be disposed after the expiry	597 [68%]
All medications must be disposed if not in use	135 [15%]
Medications should not be disposed but return to the pharmacy	137 [16%]
Other	4 [0.5%]

Data are presented as number [%], *denoted that the question may had more than one answer, hence total % may not be added to 100.

Seventy-five percent of respondents recommend that delivering clear instructions while patients receive medications raise community awareness regarding medication disposal. But 63% recommend social media as an effective tool to raise community awareness. According to the participants’ perspective, 49% stated that returning medication to a pharmacy is the best effective method to dispose of it, while 48% believed that throwing it in the household trash is the best one.

When it comes to getting rid of unused or expired medications, 68% of the participants responded that all medications should be disposed of once they have expired. Only 15% of the participants believed that all medications should be disposed of if they are not being used.

It is evident that 60% of the respondents consider pharmacies to be the primary sector responsible for this task. The results indicate that 75% of the respondents suggest providing patients with clear instructions as an effective approach to raise community awareness. The majority [88%] dispose unused medicines by throw it away in dustbins. The results reveal that 68% of the survey respondents disposed of their medication after use, whereas 15% only disposed the medications if not in use.

### Association between storage, and disposal practices of unused medicines and demographics of the survey participants

Table 4 demonstrates that there is a significant but weak association [p < 0.05; φc = 0.1], between the storage of unused medicines at home and demographic factors such as age, marital status, education, and income. However, no significant association was found between any demographic factors and the reasons for storing medicines at home [p > 0.05]. Additionally, all demographic data, with the exception of gender and residency, weakly correlate with knowledge of proper methods for storing medicines at home [p < 0.05; φc = 0.1], both age and gender are weakly associated with reading the storage instructions on the labels/ leaflets [p < 0.05, φc = 0.1], both age and gender exhibit a weak association with reading the storage instructions on medication labels/leaflets [p < 0.05, φc = 0.1]. Moreover, gender, age, and income show a significant association with the place where unused medications are stored [p < 0.05]. Additionally, all demographic factors display a weak correlation with the disposal of unused medications kept at home [p < 0.05]. The method of disposal of unused medicines also exhibits a weak association with age and marital status [p < 0.05; φc = 0.1].

**Table 4:** Association between storage, and disposal practices of unused medicines and demographics of the survey participants.

	P value						
	Gender	Age	Marital status	Residency	Education	Family size	Income
**Do you currently have any unused medicines stored at home?**	0.3	**0.01**	**<0.001**	0.8	**0.01**	**0.015**	**0.01**
	φc = N/A	**Φc = 0.13**	**Φc = 0.10**	φc = N/A	**Φc = 0.09**	**Φc = 0.01**	**Φc = 0.12**
**Why do you keep unused medicines at home?**	**0.5**	**0.3**	**0.06**	**0.6**	**0.9**	0.8	**0.7**
	φc = N/A	φc =N/A	φc = N/A	φc = N/A	φc = N/A	φc = N/A	φc =N/A
**Do you know how to store medicines at home?**	0.9	**<0.001**	**<0.001**	0.5	**0.02**	0.2	**0.04**
	φc = N/A	**Φc = 0.17**	**Φc = 0.02**	φc = N/A	**Φc = 0.1**	φc = N/A	**Φc = 0.1**
**Do you read storage instructions on the labels/leaflets?**	**0.01**	**0.04**	0.1	0.2	0.1	0.2	0.08
	**Φc = 0.09**	**Φc = 0.1**	φc = N/A	φc = N/A	φc = N/A	φc = N/A	φc = N/A
**Where do you store your unused medicine?**	**<0.001**	**0.01**	0.05	0.9	0.6	0.2	**0.014**
	**Φc = 0.29**	**Φc = 0.1**	φc = N/A	φc = N/A	φc = N/A	φc = N/A	**Φc = 0.12**
**Do you dispose of unused medicines stored at your home?**	**<.001**	**0.02**	**<.001**	0.17	**0.04**	0.3	0.7
	**Φc = 0.16**	**Φc = 0.12**	**Φc = 0.15**	φc = N/A	**Φc = 0.1**	φc = N/A	φc = N/A
**How do you dispose of unused medicines stored at your home**	**0.5**	**<0.001**	**0.012**	**0.9**	**0.4**	0.2	0.2
	φc = N/A	**0.17**	**0.16**	φc = N/A	φc = N/A	φc = N/A	φc = N/A

P value calculated by chi-square test, φc = Phi/Cramer’s V; N/A = Not applicable, P < 0.05 significant.

### Association between storage, and disposal practices of expired medicines and demographics

Table 5 demonstrates that there is a significant but weak association between check the expiry date of the medicines before purchasing and demographic factors such as age, and income [p < 0.05; φc = 0.26] and [p < 0.05; φc = 0.13] respectively, age and marital status shows weak association with check the expiry date before using the medicines [p < 0.05; φc = 0.1], only the age is associated with the method of disposal the expired and the nearly expired medication [p = 0.005;; φc = 0.1 and <0.001;; φc = 0.23 respectively}, the age and marital status are weakly associated with the knowledge of disposal of the expired medicine [p = 0.005; φc = 0.1 and 0.01; φc = 0.09 respectively].

**Table 5:** Association between storage, and disposal practices of expired medicines and demographics.

		P value					
	Gender	Age	Marital status	Residency	Education	Family size	Income
**Do you check the expiry date of the medicines before purchasing??**	**0.09**	<0.001*	<0.001*	**0.2**	**0.6**	**0.2**	0.007*
	φc =N/A	**Φc = 0.26**	**Φc = 0.22**	φc =N/A	φc =N/A	φc =N/A	**0.137**
**Do you check the expiry date before using the medicines?**	0.1	<0.001*	<0.001*	0.8	0.1	0.1	0.5
	φc =N/A	**Φc = 0.16**	**Φc = 0.15**	φc = N/A	φc = N/A	φc =N/A	φc =N/A
**How do you dispose of nearly expired medicines?**	**0.4**	<0.001*	**0.5**	**0.09**	**0.7**	**0.6**	**0.3**
	**φc = N/A**	**0.23**	**φc = N/A**	φc = N/A	φc = N/A	φc =N/A	φc =N/A
**Do you know the procedure to dispose of expired medicines?**	**0.6**	0.005*	0.01*	**0.9**	**0.3**	**0.06**	**0.2**
	**φc = N/A**	**0.14**	**0.09**	φc = N/A	φc = N/A	φc = N/A	φc =N/A
**How do you dispose of expired medicines?**	**0.7**	0.005*	**0.06**	**0.4**	**0.4**	**0.6**	**0.4**
	**φc = N/A**	**0.1**	**φc = N/A**	φc = N/A	φc = N/A	φc = N/A	φc =N/A

P value calculated by chi-square test, φc = Phi/Cramer’s V; N/A = Not applicable, P < 0.05 significant.

## Discussion

The results of this cross-sectional study provided valuable insights into the awareness and practices of individuals in Saudi Arabia about the storage and disposal of unused and expired medications. The findings highlight the need for improved education and awareness on proper medication disposal practices to ensure public health and environmental safety.

The results of this study indicate that a significant proportion (70%) of the participants had unused medications at home which is something similar to what has been reported previously [21]. According to our research, refrigerators are the primary location for storing medications in households followed by bedroom cabinets. This finding is consistent with previous studies conducted in SA [22,23]. Other study has reported different findings, where most of the respondents stored their medications in medicine cabinet [17]. To ensure the proper storage of refrigerated medications, a temperature range of 2–8 °C is necessary to prevent rapid degradation [24]. Improper storage of medications in Saudi Arabia, where summer temperatures can reach 43°C inland and 38°C at the coast, can negatively affect drug stability and efficacy.

The primary reasons for keeping unused medications included the possibility of reusing them and the need for emergencies. This highlights the importance of improving public awareness about the potential risks associated with using expired or improperly stored medications, such as reduced efficacy and potential adverse effects [25]. When it comes to medication disposal, most participants [88%] reported disposing of unused medications in the household trash. Similar findings have been reported in other studies, Serbia [80.3%] and Nigeria [70.5%] [26,27]. However, we found that the preferred method to dispose of the expired medications is also throwing away the medications in the dustbins [92%]. Studies were conducted in Afghanistan [77.7%] and Nepal [64.3%] unfortunately shows high percentages [3,19]. This practice is concerning, as it can lead to environmental contamination and potential public health risks [28]. However, one potential challenge with returning medications is that patients may be hesitant to return medications, especially if they have paid for them out of pocket. Patients may also be unsure of how to return medications or may not be aware that it is an option. Furthermore, only less than half of the participants were aware of the proper procedure for disposing of expired medications. This lack of awareness underscores the need for targeted educational interventions to promote safe medication disposal practices.

The study found that the highest proportion of participants had not received education on the safe disposal of medications, and more than half believed that pharmacies were the primary sector responsible for providing this information. This finding aligns with previous research, which has also identified pharmacies as key stakeholders in educating the public about safe medication disposal practices [18]. In addition, three quarters of our participants suggested that providing clear instructions during medication dispensing could effectively raise community awareness regarding medication disposal. This highlights the potential role of healthcare professionals, such as pharmacists and physicians, in promoting proper medication disposal practices among the public. The study also found that more than half of participants believed that all medications should be disposed of after their expiry date, while only few of our participants thought that unused medications should be disposed of regardless of their use. This finding suggests that further efforts are needed to educate the public about the potential risks associated with keeping unused and expired medications at home, as well as the importance of proper disposal practices.

In our study, we found a significant association between income and the storage of unused medication. This finding is consistent with some previous studies, such as a study conducted in the Jordan that found that Low-income families tend to procure medications in smaller quantities upon prescription refills, leading to a reduced number of stored medications within their households, indicating a positive association between income and medication storage practices [17]. Family size was also associated with the reason for storing unused medicines. The chance of storing a lot of pharmaceuticals in SA may increase when family members are more than four same residences [29]. Our study is findings align with previous research conducted in Jordan, where a significant correlation was observed between family size and the amount of unused medication stored in households [17]. Furthermore, a study conducted in Saudi Arabia revealed that larger families are more likely to practice self-medication, which could result in the purchase of unnecessary medications and the storage of unused medicines [9]. The estimated medication waste was 25.8% in the KSA A total of ~$150 million was spent by families in the KSA and other Gulf countries on medications that were never used [9]. The factor that had the strongest correlation with the increased number of medications in the home was the existence of chronic diseases in any family member. This finding is not surprising because polypharmacy is widespread in individuals with chronic illnesses. Another reason for this association may be because of different aging groups [elderly, adults, or children] taking different medication [29].

Improper disposal of unused or expired medications can lead to a variety of negative consequences, including environmental contamination, accidental ingestion, and drug abuse [30]. Saudi Vision 2030 aims to protect the natural environment to create a more diversified and sustainable economy since its strategic location that connects three continents: Africa, Asia and Europe. The medications may lead to the soil [especially for Al-Qassim’s cities nature] and seas water [specifically for coastal cities of Saudi Arabia] causing harmful effects to the environment [31]. According to a study done in Saudi Arabia, Al-Hassa region, irrigation network and its shallow lakes, the study concluded that Pharmaceuticals such as carbendazim, atorvastatin, caffeine, etoricoxib, lorazepam, metformin, paracetamol, salicylic acid, and tramadol were in 100% of the water samples [32]. To prevent these risks, the FDA recommends that most types of medications, both prescription and over the counter, be disposed of at drug take back sites or programs, which are typically located at pharmacies or hospitals [33]. These programs provide a safe and convenient way for individuals to dispose of their medications. The issue of medication waste and its associated risks have prompted many countries to implement drug take-back programs, which provide safe and convenient methods for the disposal of unused medications. In the United States, the Drug Enforcement Administration [DEA] Sponsors National Prescription Drug Take-Back Day events, and many pharmacies and hospitals have year-round take-back programs [30,33]. These programs aim to reduce medication waste and promote safe and responsible disposal practice. However, in areas where these facilities are not available or accessible, the FDA recommends safe disposal at home, with certain precautions [34]. Potentially dangerous medications such as opioids may be flushed down in the toilet to prevent accidental or intentional ingestion, misuse, or abuse. This is particularly important as these medications have a high potential for misuse and abuse, and even a single dose taken inappropriately can result in death [30]. Non-flush list medicines can be disposed of in the trash by mixing them with an unappealing substance such as dirt cat litter, or coffee grounds; placing the mixture in a sealed plastic bag; and throwing it in the trash. It is important to note that empty medicine bottles should be disposed of separately, either in the trash, recycled, or packaged, to prevent accidental ingestion or misuse [35]. By following these guidelines, individuals can help ensure the safe and responsible disposal of unused or expired medications. Improper disposal of all dosage forms is dangerous, especially for the injectable ones [et. contain needle or syringe as an important component for their route of administration] specifically if they disposed by throwing them in the dustbin [the most disposal method shown in the study].

### Limitations

The predominance of female, urban, and educated respondents is likely a consequence of online convenience sampling, which may over-represent educated urban women. The possibility of selection, reporting and recall bias cannot be overlooked due to the cross-sectional study design. Refrigerator storage reported in this study refers specifically to unused medications; therefore, it should not be interpreted as storage of medicines that require refrigeration during active use.

## Conclusions

This study highlights the need for improved education and awareness regarding the storage and disposal of unused and expired medications in Saudi Arabia. Healthcare professionals, particularly pharmacists, can play a crucial role in promoting safe medication disposal practices among the public. Future interventions should focus on providing clear instructions on drug disposal during medication dispensing and leveraging social media to raise community awareness of the potential risks associated with improper medication disposal. Moreover, the implementation of designated medication disposal locations such as community pharmacy-based take back centers and smartphone applications may facilitate safe medication waste management practices. Furthermore, the policy makers and Ministry of Health can initiate the drug disposal plan regionally and countrywide to ensure proper disposal of unused medicines.

We recommend that future research build on our findings by extending the data collection period beyond two months to capture potential seasonal variations in medication disposal practices. Additionally, expanding the online questionnaire to a larger and more diverse population across different regions in Saudi Arabia which our study currently lacks, would also help provide a more comprehensive understanding of public awareness and behaviors.

## Supporting information

S1 File Researchers reflexive positioning. (DOCX)

## Abbreviations

EPV: Echopharmacovigilance
PV: Pharmacovigilance
KSA: Kingdom of Saudi Arabia
OTC: Over the counter
USA: United states of America
WHO: The world health organization
CSCW: Computer Supported Collaborative Work
