# Supplementary material for: Addressing the disposal of unused and expired medications in Saudi households: Time to bridge the regulatory gaps
Source: PLoS One. 2026 Jun 16;21(6):e0350117. doi: 10.1371/journal.pone.0350117 (PMC13271494; doi:10.1371/journal.pone.0350117)
Supplement: S1 File — (DOCX) [file pone.0350117.s001.docx]

**S1 Text. Researcher Reflexive positioning**

The primary data analysis was conducted by a research team of Saudi and Malaysian healthcare professionals. The lead analyst (MF), a pharmacy graduate with an interest in social pharmacy and public health, brought an 'insider' perspective to the study as a resident of the Qassim region. Having observed the common household practice of storing large quantities of unused medications among peers and family members, the researcher was motivated to investigate the underlying motivations and regulatory failures contributing to this behavior. At the time of data collection, the research team included final-year pharmacy students (AA, DA, RA, RA) and established academics (SA, WII, SA), ensuring a balance between fresh clinical perspectives and experienced methodological oversight. The researchers hold a professional interest in medication safety, harm reduction, and environmental protection, often volunteering in community health programs to reduce the risks of accidental poisoning. None of the researchers had prior personal relationships with the participants, ensuring that the findings remained objective while benefiting from the team's deep cultural and professional familiarity with the Saudi healthcare context."

Maryam Farooqui (MF)

Aseel Alrashed,

Duaa Alfneekh (DA)

Raffal Alassaf (RA)

Reem Aldubayan (RA)

Saud Alsahali (SA)

Wan Ismahanisa Ismail (WII)

Suhaj Abdulsalim (SA)
