# Supplementary material for: Addressing the disposal of unused and expired medications in Saudi households: Time to bridge the regulatory gaps
Source: PLoS One. 2026 Jun 16;21(6):e0350117. doi: 10.1371/journal.pone.0350117 (PMC13271494; doi:10.1371/journal.pone.0350117)
Supplement: Attachment — Submitted filename: Rebuttle 20 april.docx [file pone.0350117.s004.docx]

5. Review Comments to the Author

Reviewer #1: The manuscript is well written but still has some areas to be improved in the introduction and discussion section. The statistical results are not sure to me, hence i would recommend to confirm about them.

Reviewer #2: The topic is important and suitable for PLOS ONE because improper household storage and disposal of medicines can have public health and environmental implications, and the paper addresses a policy-relevant gap in Saudi Arabia. However, i have some comments:

1. The design is described inconsistently, and you should correct the study design label everywhere: the manuscript repeatedly calls this a “prospective cross-sectional survey,” which is internally contradictory terminology for a one-time questionnaire survey. Please revise to “cross-sectional survey” unless you truly followed respondents over time (which is not described).

**Thank you for pointing this out. We agree that the term ‘prospective cross-sectional survey’ was inappropriate. The study design has now been corrected to ‘cross-sectional survey’ consistently throughout the manuscript, as data were collected at a single time point with no follow-up of respondents. Abstract (Page 2: Line 35); Keyword: (page 2, line 62); Study design and Population (Page 4, line 127); Discussion (Page 14, line 287); Limitations (Page 16, line 402)**

2. The data collection period is unclear and inconsistent across sections, and this must be reconciled: the front matter/abstract describes a “three-month period,” while the Methods state data collection from 20 December 2022 to 28 February 2023 (about ~2–2.5 months). Please make the period consistent across Abstract, Methods, and Results.

**Thank you for highlighting this inconsistency. We have now clarified and fixed the data collection period across all sections of the manuscript. The data collection period has been consistently stated as 20 December 2022 to 28 February 2023 in the Abstract, Methods, and Results sections. Study design and Population (Page 4, line 128)**

3. The sampling approach is not transparent enough to evaluate bias: you state “proportional sampling was adopted,” but data were collected via an online questionnaire without describing any sampling frame, recruitment channels, strata, or allocation procedure. Please specify exactly how proportional sampling was implemented (what strata, how proportions were determined, how participants were reached, and how duplicates were prevented).

**Thank you for this comment. We acknowledge that the sampling method used was convenience sampling rather than proportional sampling. The Methods section has been revised accordingly to accurately describe the recruitment approach. Participants were recruited through online dissemination of the questionnaire via personal and institutional networks. Study design and Population (Page 4, line 131-133)**

4. The sample profile strongly suggests selection bias and limited generalisability, and you should address this explicitly and (if possible) analytically: 81% female, 98% urban, and 77% graduates is unlikely to reflect the region’s adult population distribution, especially if the stated target population is all Al-Qassim residents. At minimum, acknowledge that the online recruitment likely over-represented educated urban women; ideally, compare key demographics to official population distributions and discuss implications for inference.

**Thank you for this important observation. We agree that the sample profile indicates potential selection bias and limited generalisability. The predominance of female, urban, and highly educated respondents is likely a consequence of online convenience sampling, which may over-represent educated urban women. We have now explicitly acknowledged this limitation in the Discussion section and clarified that the findings should be interpreted with caution. Limitations (Page 16, line 399-402)**

5. The eligibility criteria need tightening and justification: the requirement “psychologically stable” is vague, ethically sensitive, and not operationalised in an online survey context. Please remove it or define how it was assessed (and why it was necessary).

**Thanks, we totally agree with this concern, and it has been removed from the text. (Page 4, Line 167)**

6. The questionnaire reliability reporting appears incorrect and must be corrected because it affects confidence in all results: you report Cronbach’s alpha as 0.08 and state it is acceptable, which would normally indicate extremely poor internal consistency. This is likely a typo (e.g., 0.80) or a misapplied statistic (alpha is not meaningful for mixed-domain checklists). Please (a) confirm the correct alpha value, (b) specify which items formed the scale used for alpha, and (c) report reliability separately for subscales if applicable.

**Very sorry, it was a typo and we rectified. Thanks (Page 4, line 185)**

7. The instrument description is incomplete for reproducibility: you describe a 64-item tool and cite appendices (Appendix B, Appendix D), but the manuscript should ensure the full questionnaire (Arabic and/or English) is provided in Supporting Information, with scoring rules and skip logic.

**It was an inadvertent error; the appendix information was mistakenly added. The questionnaire has been provided as a supplementary file now. (Page 4, line 179)**

8. Several key percentages are hard to interpret because denominators are not always explicit, especially where responses are conditional or “select all that apply”: for example, storage location percentages appear to be among those reporting unused medicines, while other outcomes appear to be among all respondents. Please state denominators clearly in each table and in the text (e.g., “among those with unused medicines, n=612…”).

**A footnote has been added to Table 2 to make it clearer. (Page 9)**

9. There is a major internal contradiction about who respondents think is responsible for public education: the Abstract states that 60% “denied that the responsible sector… should be pharmacies,” yet Table 3 lists pharmacies as a responsible sector selected by 60%, and the text under Figure 1 also states 60% consider pharmacies primary. This must be resolved by checking the exact wording/coding of the question and rewriting the Results and Abstract accordingly (e.g., was the item “pharmacies are responsible—yes/no,” or “which sectors are responsible—select all”?)

**Thanks for notifying this mistake, yes, it was a typo where 0% agrees that pharmacies should educate people on safe disposal of medicines. and the abstract has been updated now. (Page 2, line 53)**

10. The statistical analysis plan needs improvement in three specific ways: (a) you rely heavily on chi-square tests with a large sample where small differences become “significant,” (b) you report weak effect sizes (e.g., φc≈0.1) but do not interpret them properly, and (c) you run many tests across many demographics without addressing multiple comparisons. Please focus interpretation on effect sizes and pre-specified hypotheses, and consider adjusting for multiple testing (or explicitly justify not doing so).

**Thanks for the comments. When we went through other literature and previous studies, we want to make different comparison and association. Hence, we did accordingly.**

11. The tables reporting p-values and effect sizes are difficult to read and in places appear misformatted (e.g., split φc values across lines), and should be cleaned for readability and accuracy. Please ensure each cell clearly shows p-value and effect size, or separate them into two clean columns, and ensure the same effect size metric is used consistently (Cramér’s V is typical for r×c).

**The tables have been formatted**

12. Several variable labels and text contain errors that affect clarity and should be corrected before review can focus on substance (examples include income band text “More than 5000 SR-1000SR,” repeated/duplicated sentences, and multiple grammar issues such as “which smoothing similar”). A thorough language edit is needed.

**The manuscript has undergone a thorough language revision to correct grammatical issues, improve clarity. Monthly income band description have been corrected to ensure accurate interpretation.**

13. The Results presentation would be stronger if you add one consolidated summary table (or figure) that shows, at a minimum: prevalence of unused medicines, top storage locations, top disposal methods for unused/near-expiry/expired medicines, and awareness indicators, each with clear denominators and 95% confidence intervals. Right now results are scattered across multiple tables and narrative.

**Table 2 and 3 are explaining the above said information. If we add another table, it will be just repeating of information**.

14. The interpretation of “refrigerator storage” needs more nuance: you report that 62% store unused medicines in the refrigerator, but you do not assess whether those medicines actually require refrigeration or whether refrigeration is appropriate (many medicines should not be refrigerated, and temperature control in domestic fridges varies). If you did not collect medicine type/formulation, acknowledge that you cannot judge appropriateness; if you did, stratify by “requires refrigeration” vs “does not.”

**Thank you for this comment. In our study, the question referred specifically to the storage location of unused medications, which differs from medications that require refrigeration during active use (e.g., insulin or certain biologics). Our intention was to identify common household practices for storing leftover or unused medicines rather than to assess the appropriateness of storage conditions for medicines currently in use. As information on the type or formulation of the unused medicines was not collected, we are unable to determine whether refrigeration was necessary. This clarification has been added in the limitation section. (Page 16, line 400)**

15. Several environmental/AMR claims are phrased too strongly relative to what this study can support: for example, the Introduction suggests improper disposal may “increase medication resistance,” and the Discussion expands to wide claims about worldwide harm and flushing guidance. Please keep causal language proportionate—this study measures knowledge and self-reported practices, not environmental contamination or AMR outcomes—and ensure all such claims are tightly referenced and accurately worded (e.g., “may contribute to antimicrobial selection pressure” rather than asserting it does).

**Updated as suggested. (Page 14)**

16. The ethical procedures need slightly more detail for an online survey: you state ethics approval and informed consent, but please explain how consent was obtained online (checkbox, information sheet, ability to withdraw) and whether any personal identifiers were collected.

**Updated as suggested (Page 5, line 185)**

17. The manuscript should report missing data handling and data cleaning steps (e.g., whether incomplete questionnaires were excluded, how “Not sure” responses were handled in denominators, and whether any logic checks were applied), because this affects the validity of the reported percentages.

**Updated as suggested under the heading “Data analysis”. (Page 6, Line 167-177)**

18. The conclusion and recommendations should be made more specific and actionable: you recommend “regulatory guidelines” and “awareness,” but the paper can do better by translating the findings into concrete interventions (e.g., pharmacy-based take-back pilots in Qassim, standardized patient-facing disposal instructions at dispensing, national guidance on what should/should not be flushed, and evaluation plans). This can be tied directly to the large proportion reporting disposal in household bins and low formal education exposure.

**Modified as per comments (Page 19-20 line 432-449)**

19 The limitations section is on the right track but should explicitly list the biggest threats to validity in this study: convenience online sampling, non-representative demographics, self-report and social desirability bias, inability to establish temporality/causality, and inability to verify actual disposal behaviors.

**Updated as suggested. (Page 19, Line 421-430)**
