# Supplementary material for: Addressing the disposal of unused and expired medications in Saudi households: Time to bridge the regulatory gaps
Source: PLoS One. 2026 Jun 16;21(6):e0350117. doi: 10.1371/journal.pone.0350117 (PMC13271494; doi:10.1371/journal.pone.0350117)
Supplement: Peer review history

# Peer review: Decision Letter 0

**DOI:** 10.1371/journal.pone.0350117.r001

**Authors:** Dawit Getachew Gebeyehu

20 Jan 2026

PONE-D-25-52812

Addressing the Disposal of Unused and Expired Medications in Saudi Households: Time to bridge the regulatory gaps

PLOS One

Dear Dr. Abdulsalim,

Thank you for submitting your manuscript to PLOS ONE. After careful consideration, we feel that it has merit but does not fully meet PLOS ONE’s publication criteria as it currently stands. Therefore, we invite you to submit a revised version of the manuscript that addresses the points raised during the review process.

Please submit your revised manuscript by Mar 06 2026 11:59PM. If you will need more time than this to complete your revisions, please reply to this message or contact the journal office at plosone@plos.org. When you're ready to submit your revision, log on to https://www.editorialmanager.com/pone/ and select the 'Submissions Needing Revision' folder to locate your manuscript file.

Please include the following items when submitting your revised manuscript:

- A letter that responds to each point raised by the academic editor and reviewer(s). You should upload this letter as a separate file labeled 'Response to Reviewers'.
- A marked-up copy of your manuscript that highlights changes made to the original version. You should upload this as a separate file labeled 'Revised Manuscript with Track Changes'.
- An unmarked version of your revised paper without tracked changes. You should upload this as a separate file labeled 'Manuscript'.

If you would like to make changes to your financial disclosure, please include your updated statement in your cover letter. Guidelines for resubmitting your figure files are available below the reviewer comments at the end of this letter.

If applicable, we recommend that you deposit your laboratory protocols in protocols.io to enhance the reproducibility of your results. Protocols.io assigns your protocol its own identifier (DOI) so that it can be cited independently in the future. For instructions see: https://journals.plos.org/plosone/s/submission-guidelines#loc-laboratory-protocols. Additionally, PLOS ONE offers an option for publishing peer-reviewed Lab Protocol articles, which describe protocols hosted on protocols.io. Read more information on sharing protocols at https://plos.org/protocols?utm_medium=editorial-email&utm_source=authorletters&utm_campaign=protocols.

We look forward to receiving your revised manuscript.

Kind regards,

Dawit Getachew Gebeyehu, MPH

Academic Editor

PLOS One

Journal Requirements:

When submitting your revision, we need you to address these additional requirements.

1. Please ensure that your manuscript meets PLOS ONE's style requirements, including those for file naming. The PLOS ONE style templates can be found at https://journals.plos.org/plosone/s/file?id=wjVg/PLOSOne_formatting_sample_main_body.pdf and https://journals.plos.org/plosone/s/file?id=ba62/PLOSOne_formatting_sample_title_authors_affiliations.pdf

2. Your ethics statement should only appear in the Methods section of your manuscript. If your ethics statement is written in any section besides the Methods, please delete it from any other section.

3. Thank you for stating the following in the Acknowledgments Section of your manuscript:

The researchers would like to thank the Deanship of Graduate Studies and Scientific Research at Qassim University for financial support (APC-QU- (2025). The authors would also like to express their gratitude to all participants of this study.

We note that you have provided funding information that is not currently declared in your Funding Statement. However, funding information should not appear in the Acknowledgments section or other areas of your manuscript. We will only publish funding information present in the Funding Statement section of the online submission form.

Please remove any funding-related text from the manuscript and let us know how you would like to update your Funding Statement. Currently, your Funding Statement reads as follows:

The author(s) received no specific funding for this work.

Please include your amended statements within your cover letter; we will change the online submission form on your behalf.

4. We note that Figure(s) 1, 2, 3, 4, 5, in your submission contain copyrighted images. All PLOS content is published under the Creative Commons Attribution License (CC BY 4.0), which means that the manuscript, images, and Supporting Information files will be freely available online, and any third party is permitted to access, download, copy, distribute, and use these materials in any way, even commercially, with proper attribution. For more information, see our copyright guidelines: http://journals.plos.org/plosone/s/licenses-and-copyright.

We require you to either (1) present written permission from the copyright holder to publish these figures specifically under the CC BY 4.0 license, or (2) remove the figures from your submission:

a. You may seek permission from the original copyright holder of Figure(s) 1, 2, 3, 4, 5, to publish the content specifically under the CC BY 4.0 license.

We recommend that you contact the original copyright holder with the Content Permission Form (http://journals.plos.org/plosone/s/file?id=7c09/content-permission-form.pdf) and the following text:

“I request permission for the open-access journal PLOS ONE to publish XXX under the Creative Commons Attribution License (CCAL) CC BY 4.0 (http://creativecommons.org/licenses/by/4.0/). Please be aware that this license allows unrestricted use and distribution, even commercially, by third parties. Please reply and provide explicit written permission to publish XXX under a CC BY license and complete the attached form.”

Please upload the completed Content Permission Form or other proof of granted permissions as an "Other" file with your submission.

In the figure caption of the copyrighted figure, please include the following text: “Reprinted from [ref] under a CC BY license, with permission from [name of publisher], original copyright [original copyright year].”

b. If you are unable to obtain permission from the original copyright holder to publish these figures under the CC BY 4.0 license or if the copyright holder’s requirements are incompatible with the CC BY 4.0 license, please either i) remove the figure or ii) supply a replacement figure that complies with the CC BY 4.0 license. Please check copyright information on all replacement figures and update the figure caption with source information. If applicable, please specify in the figure caption text when a figure is similar but not identical to the original image and is therefore for illustrative purposes only.

5. Please ensure that you refer to Figure 1, 2, 3, 4, 5, in your text as, if accepted, production will need this reference to link the reader to the figure.

6. If the reviewer comments include a recommendation to cite specific previously published works, please review and evaluate these publications to determine whether they are relevant and should be cited. There is no requirement to cite these works unless the editor has indicated otherwise.

[Note: HTML markup is below. Please do not edit.]

Reviewers' comments:

Reviewer's Responses to Questions

**Comments to the Author**

1. Is the manuscript technically sound, and do the data support the conclusions?

The manuscript must describe a technically sound piece of scientific research with data that supports the conclusions. Experiments must have been conducted rigorously, with appropriate controls, replication, and sample sizes. The conclusions must be drawn appropriately based on the data presented.

Reviewer #1: Yes

Reviewer #2: Yes

**********

2. Has the statistical analysis been performed appropriately and rigorously?

Reviewer #1: I Don't Know

Reviewer #2: Yes

**********

3. Have the authors made all data underlying the findings in their manuscript fully available?

The PLOS Data policy requires authors to make all data underlying the findings described in their manuscript fully available without restriction, with rare exception (please refer to the Data Availability Statement in the manuscript PDF file). The data should be provided as part of the manuscript or its supporting information, or deposited to a public repository. For example, in addition to summary statistics, the data points behind means, medians and variance measures should be available. If there are restrictions on publicly sharing data—e.g. participant privacy or use of data from a third party—those must be specified.

Reviewer #1: No

Reviewer #2: No

**********

4. Is the manuscript presented in an intelligible fashion and written in standard English?

PLOS ONE does not copyedit accepted manuscripts, so the language in submitted articles must be clear, correct, and unambiguous. Any typographical or grammatical errors should be corrected at revision, so please note any specific errors here.

Reviewer #1: No

Reviewer #2: Yes

**********

5. Review Comments to the Author

Please use the space provided to explain your answers to the questions above. You may also include additional comments for the author, including concerns about dual publication, research ethics, or publication ethics. (Please upload your review as an attachment if it exceeds 20,000 characters)

Reviewer #1: The manuscript is well written but still has some areas to be improved in the introduction and discussion section. The statistical results are not sure to me, hence i would recommend to confirm about them.

Reviewer #2: The topic is important and suitable for PLOS ONE because improper household storage and disposal of medicines can have public health and environmental implications, and the paper addresses a policy-relevant gap in Saudi Arabia. However, i have some comments:

1. The design is described inconsistently, and you should correct the study design label everywhere: the manuscript repeatedly calls this a “prospective cross-sectional survey,” which is internally contradictory terminology for a one-time questionnaire survey. Please revise to “cross-sectional survey” unless you truly followed respondents over time (which is not described).

2. The data collection period is unclear and inconsistent across sections, and this must be reconciled: the front matter/abstract describes a “three-month period,” while the Methods state data collection from 20 December 2022 to 28 February 2023 (about ~2–2.5 months). Please make the period consistent across Abstract, Methods, and Results.

3. The sampling approach is not transparent enough to evaluate bias: you state “proportional sampling was adopted,” but data were collected via an online questionnaire without describing any sampling frame, recruitment channels, strata, or allocation procedure. Please specify exactly how proportional sampling was implemented (what strata, how proportions were determined, how participants were reached, and how duplicates were prevented).

4. The sample profile strongly suggests selection bias and limited generalisability, and you should address this explicitly and (if possible) analytically: 81% female, 98% urban, and 77% graduates is unlikely to reflect the region’s adult population distribution, especially if the stated target population is all Al-Qassim residents. At minimum, acknowledge that the online recruitment likely over-represented educated urban women; ideally, compare key demographics to official population distributions and discuss implications for inference.

5. The eligibility criteria need tightening and justification: the requirement “psychologically stable” is vague, ethically sensitive, and not operationalised in an online survey context. Please remove it or define how it was assessed (and why it was necessary).

6. The questionnaire reliability reporting appears incorrect and must be corrected because it affects confidence in all results: you report Cronbach’s alpha as 0.08 and state it is acceptable, which would normally indicate extremely poor internal consistency. This is likely a typo (e.g., 0.80) or a misapplied statistic (alpha is not meaningful for mixed-domain checklists). Please (a) confirm the correct alpha value, (b) specify which items formed the scale used for alpha, and (c) report reliability separately for subscales if applicable.

7. The instrument description is incomplete for reproducibility: you describe a 64-item tool and cite appendices (Appendix B, Appendix D), but the manuscript should ensure the full questionnaire (Arabic and/or English) is provided in Supporting Information, with scoring rules and skip logic.

8. Several key percentages are hard to interpret because denominators are not always explicit, especially where responses are conditional or “select all that apply”: for example, storage location percentages appear to be among those reporting unused medicines, while other outcomes appear to be among all respondents. Please state denominators clearly in each table and in the text (e.g., “among those with unused medicines, n=612…”).

9. There is a major internal contradiction about who respondents think is responsible for public education: the Abstract states that 60% “denied that the responsible sector… should be pharmacies,” yet Table 3 lists pharmacies as a responsible sector selected by 60%, and the text under Figure 1 also states 60% consider pharmacies primary. This must be resolved by checking the exact wording/coding of the question and rewriting the Results and Abstract accordingly (e.g., was the item “pharmacies are responsible—yes/no,” or “which sectors are responsible—select all”?)

10. The statistical analysis plan needs improvement in three specific ways: (a) you rely heavily on chi-square tests with a large sample where small differences become “significant,” (b) you report weak effect sizes (e.g., ϕc≈0.1) but do not interpret them properly, and (c) you run many tests across many demographics without addressing multiple comparisons. Please focus interpretation on effect sizes and pre-specified hypotheses, and consider adjusting for multiple testing (or explicitly justify not doing so).

11. The tables reporting p-values and effect sizes are difficult to read and in places appear misformatted (e.g., split ϕc values across lines), and should be cleaned for readability and accuracy. Please ensure each cell clearly shows p-value and effect size, or separate them into two clean columns, and ensure the same effect size metric is used consistently (Cramér’s V is typical for r×c).

12. Several variable labels and text contain errors that affect clarity and should be corrected before review can focus on substance (examples include income band text “More than 5000 SR-1000SR,” repeated/duplicated sentences, and multiple grammar issues such as “which smoothing similar”). A thorough language edit is needed.

13. The Results presentation would be stronger if you add one consolidated summary table (or figure) that shows, at a minimum: prevalence of unused medicines, top storage locations, top disposal methods for unused/near-expiry/expired medicines, and awareness indicators, each with clear denominators and 95% confidence intervals. Right now results are scattered across multiple tables and narrative.

14. The interpretation of “refrigerator storage” needs more nuance: you report that 62% store unused medicines in the refrigerator, but you do not assess whether those medicines actually require refrigeration or whether refrigeration is appropriate (many medicines should not be refrigerated, and temperature control in domestic fridges varies). If you did not collect medicine type/formulation, acknowledge that you cannot judge appropriateness; if you did, stratify by “requires refrigeration” vs “does not.”

15. Several environmental/AMR claims are phrased too strongly relative to what this study can support: for example, the Introduction suggests improper disposal may “increase medication resistance,” and the Discussion expands to wide claims about worldwide harm and flushing guidance. Please keep causal language proportionate—this study measures knowledge and self-reported practices, not environmental contamination or AMR outcomes—and ensure all such claims are tightly referenced and accurately worded (e.g., “may contribute to antimicrobial selection pressure” rather than asserting it does).

16. The ethical procedures need slightly more detail for an online survey: you state ethics approval and informed consent, but please explain how consent was obtained online (checkbox, information sheet, ability to withdraw) and whether any personal identifiers were collected.

17. The manuscript should report missing data handling and data cleaning steps (e.g., whether incomplete questionnaires were excluded, how “Not sure” responses were handled in denominators, and whether any logic checks were applied), because this affects the validity of the reported percentages.

18. The conclusion and recommendations should be made more specific and actionable: you recommend “regulatory guidelines” and “awareness,” but the paper can do better by translating the findings into concrete interventions (e.g., pharmacy-based take-back pilots in Qassim, standardized patient-facing disposal instructions at dispensing, national guidance on what should/should not be flushed, and evaluation plans). This can be tied directly to the large proportion reporting disposal in household bins and low formal education exposure.

19 The limitations section is on the right track but should explicitly list the biggest threats to validity in this study: convenience online sampling, non-representative demographics, self-report and social desirability bias, inability to establish temporality/causality, and inability to verify actual disposal behaviors.

**********

6. PLOS authors have the option to publish the peer review history of their article (what does this mean?). If published, this will include your full peer review and any attached files.

If you choose “no”, your identity will remain anonymous but your review may still be made public.

**Do you want your identity to be public for this peer review?**  For information about this choice, including consent withdrawal, please see our Privacy Policy.

Reviewer #1: No

Reviewer #2: **Yes:** Yusuff Adebayo Adebisi

**********

[NOTE: If reviewer comments were submitted as an attachment file, they will be attached to this email and accessible via the submission site. Please log into your account, locate the manuscript record, and check for the action link "View Attachments". If this link does not appear, there are no attachment files.]

To ensure your figures meet our technical requirements, please review our figure guidelines: https://journals.plos.org/plosone/s/figures

You may also use PLOS’s free figure tool, NAAS, to help you prepare publication quality figures: https://journals.plos.org/plosone/s/figures#loc-tools-for-figure-preparation.

NAAS will assess whether your figures meet our technical requirements by comparing each figure against our figure specifications.

# Author comment: Author response to Decision Letter 1

**DOI:** 10.1371/journal.pone.0350117.r002

25 Mar 2026

we have addressed all comments in a seperate file

Attachment Submitted filename: Rebuttle- plose one.docx

# Editor report: Decision Letter 1

**DOI:** 10.1371/journal.pone.0350117.r003

**Authors:** Dawit Getachew Gebeyehu

28 Mar 2026

PONE-D-25-52812R1

Addressing the Disposal of Unused and Expired Medications in Saudi Households: Time to bridge the regulatory gaps

PLOS One

Dear Dr. Abdulsalim,

Thank you for submitting your manuscript to PLOS ONE. After careful consideration, we feel that it has merit but does not fully meet PLOS ONE’s publication criteria as it currently stands. Therefore, we invite you to submit a revised version of the manuscript that addresses the points raised during the review process.

1. Please respond to the journal requirement comment, particularly figure copyright issue raised during the first round revision

2. Provide line number in to your manuscript.

3. In your point-by-point response, add page number and line number to indicate where the change was made in the revised manuscript.

Please submit your revised manuscript by May 12 2026 11:59PM. If you will need more time than this to complete your revisions, please reply to this message or contact the journal office at plosone@plos.org. When you're ready to submit your revision, log on to https://www.editorialmanager.com/pone/ and select the 'Submissions Needing Revision' folder to locate your manuscript file.

Please include the following items when submitting your revised manuscript:

- A letter that responds to each point raised by the academic editor and reviewer(s). You should upload this letter as a separate file labeled 'Response to Reviewers'.
- A marked-up copy of your manuscript that highlights changes made to the original version. You should upload this as a separate file labeled 'Revised Manuscript with Track Changes'.
- An unmarked version of your revised paper without tracked changes. You should upload this as a separate file labeled 'Manuscript'.

If you would like to make changes to your financial disclosure, please include your updated statement in your cover letter. Guidelines for resubmitting your figure files are available below the reviewer comments at the end of this letter.

If applicable, we recommend that you deposit your laboratory protocols in protocols.io to enhance the reproducibility of your results. Protocols.io assigns your protocol its own identifier (DOI) so that it can be cited independently in the future. For instructions see: https://journals.plos.org/plosone/s/submission-guidelines#loc-laboratory-protocols. Additionally, PLOS ONE offers an option for publishing peer-reviewed Lab Protocol articles, which describe protocols hosted on protocols.io. Read more information on sharing protocols at https://plos.org/protocols?utm_medium=editorial-email&utm_source=authorletters&utm_campaign=protocols.

As the corresponding author, your ORCID iD is verified in the submission system and will appear in the published article. PLOS supports the use of ORCID, and we encourage all coauthors to register for an ORCID iD and use it as well. Please encourage your coauthors to verify their ORCID iD within the submission system before final acceptance, as unverified ORCID iDs will not appear in the published article. *Only*  the individual author can complete the verification step; PLOS staff *cannot*  verify ORCID iDs on behalf of authors.

We look forward to receiving your revised manuscript.

Kind regards,

Dawit Getachew Gebeyehu, MPH

Academic Editor

PLOS One

Journal Requirements:

If the reviewer comments include a recommendation to cite specific previously published works, please review and evaluate these publications to determine whether they are relevant and should be cited. There is no requirement to cite these works unless the editor has indicated otherwise.

Additional Editor Comments:

Dear Authors, thank you for your efforts to address the reviewer's concern; however, please include the page and line number where this change was made. Also, please address journal requirements, ensure that they are included in your point-by-point response, and upload them separately as pdfs.

[Note: HTML markup is below. Please do not edit.]

[NOTE: If reviewer comments were submitted as an attachment file, they will be attached to this email and accessible via the submission site. Please log into your account, locate the manuscript record, and check for the action link "View Attachments". If this link does not appear, there are no attachment files.]

To ensure your figures meet our technical requirements, please review our figure guidelines: https://journals.plos.org/plosone/s/figures

You may also use PLOS’s free figure tool, NAAS, to help you prepare publication quality figures: https://journals.plos.org/plosone/s/figures#loc-tools-for-figure-preparation.

NAAS will assess whether your figures meet our technical requirements by comparing each figure against our figure specifications.

# Author comment: Author response to Decision Letter 2

**DOI:** 10.1371/journal.pone.0350117.r004

25 Apr 2026

Thank you very much for your comments. We have addressed all your comments.

Attachment Submitted filename: Rebuttle 20 april.docx

# Editor report: Decision Letter 2

**DOI:** 10.1371/journal.pone.0350117.r005

**Authors:** Dawit Getachew Gebeyehu

11 May 2026

Addressing the Disposal of Unused and Expired Medications in Saudi Households: Time to bridge the regulatory gaps

PONE-D-25-52812R2

Dear Dr. Abdusalim,

We’re pleased to inform you that your manuscript has been judged scientifically suitable for publication and will be formally accepted for publication once it meets all outstanding technical requirements.

Within one week, you’ll receive an e-mail detailing the required amendments. When these have been addressed, you’ll receive a formal acceptance letter and your manuscript will be scheduled for publication.

An invoice will be generated when your article is formally accepted. Please note, if your institution has a publishing partnership with PLOS and your article meets the relevant criteria, all or part of your publication costs will be covered. Please make sure your user information is up-to-date by logging into Editorial Manager at Editorial Manager® and clicking the ‘Update My Information' link at the top of the page. For questions related to billing, please contact billing support.

If your institution or institutions have a press office, please notify them about your upcoming paper to help maximize its impact. If they’ll be preparing press materials, please inform our press team as soon as possible -- no later than 48 hours after receiving the formal acceptance. Your manuscript will remain under strict press embargo until 2 pm Eastern Time on the date of publication. For more information, please contact onepress@plos.org.

Kind regards,

Dawit Getachew Gebeyehu

Academic Editor

PLOS One

# Editor report: Acceptance letter

**DOI:** 10.1371/journal.pone.0350117.r006

**Authors:** Dawit Getachew Gebeyehu

PONE-D-25-52812R2

PLOS One

Dear Dr. Abdulsalim,

I'm pleased to inform you that your manuscript has been deemed suitable for publication in PLOS One. Congratulations! Your manuscript is now being handed over to our production team.

At this stage, our production department will prepare your paper for publication. This includes ensuring the following:

* All references, tables, and figures are properly cited

* All relevant supporting information is included in the manuscript submission,

* There are no issues that prevent the paper from being properly typeset

You will receive further instructions from the production team, including instructions on how to review your proof when it is ready. Please keep in mind that we are working through a large volume of accepted articles, so please give us a few days to review your paper and let you know the next and final steps.

Lastly, if your institution or institutions have a press office, please let them know about your upcoming paper now to help maximize its impact. If they'll be preparing press materials, please inform our press team within the next 48 hours. Your manuscript will remain under strict press embargo until 2 pm Eastern Time on the date of publication. For more information, please contact onepress@plos.org.

You will receive an invoice from PLOS for your publication fee after your manuscript has reached the completed accept phase. If you receive an email requesting payment before acceptance or for any other service, this may be a phishing scheme. Learn how to identify phishing emails and protect your accounts at https://explore.plos.org/phishing.

If we can help with anything else, please email us at customercare@plos.org.

Thank you for submitting your work to PLOS ONE and supporting open access.

Kind regards,

PLOS ONE Editorial Office Staff

on behalf of

Mr. Dawit Getachew Gebeyehu

Academic Editor

PLOS One
